# Personality, Defense Mechanisms and Psychological Distress in Women with Fibromyalgia

**DOI:** 10.3390/bs12010010

**Published:** 2022-01-07

**Authors:** Annunziata Romeo, Agata Benfante, Giuliano Carlo Geminiani, Lorys Castelli

**Affiliations:** 1 Department of Psychology, University of Turin, 10124 Turin, Italy; annunziata.romeo@unito.it (A.R.); giulianocarlo.geminiani@unito.it (G.C.G.); lorys.castelli@unito.it (L.C.); 2 Città Della Salute e Della Scienza Hospital, 10126 Turin, Italy

**Keywords:** personality traits, alexithymia, defense mechanisms, psychological distress, fibromyalgia

## Abstract

Background: Previous studies have shown that many personality traits are associated with fibromyalgia (FM), worsening both the quality of life and psychological distress of patients. Despite the high comorbidity of psychopathological disorders in this syndrome and their association with immature defense styles, few studies have examined the defense mechanisms used by FM patients. The main aim of our study was to investigate personality traits and defense mechanisms in FM patients compared to in a healthy control group (HC). Moreover, we investigated the effect of personality traits and defense mechanisms on psychological distress in both FM and HC groups. Methods: A total of 54 women with FM and 54 healthy women completed the (1) Temperament and Character Inventory—Revised; (2) the Toronto Alexithymia Scale; (3) the Defense Style Questionnaire; and (4) the Hospital Anxiety and Depression Scale. Results: The results indicated that FM patients display higher alexithymia, higher harm avoidance, lower self-directedness, lower persistence, and the higher use of a maladaptive defense style compared to HC. We found that alexithymia, harm avoidance, and maladaptive defense style are significant predictors of patients’ psychological distress. Moreover, harm avoidance and adaptive defense style significantly predicted psychological distress in the HC group. Conclusion: The present study is the first to explore the contribution of both defense mechanisms and personality characteristics on the psychological distress of FM patients. Our findings have important clinical implications and may help diagnose and treat FM patients more in depth.

## 1. Introduction

Fibromyalgia (FM) is a chronic syndrome that is characterized by widespread musculoskeletal pain [1] and has a high incidence among women [2,3]. FM is the third most prevalent musculoskeletal condition, and its etiopathogenesis is still debated due to its complexity and multi-factoriality. A series of other physical, psychological, and cognitive symptoms are often associated with FM [3]. This condition negatively affects patient quality of life and can have significant psychological and relational consequences. The literature suggests that FM patients experience excessive levels of psychological distress: 20–80% experience anxiety and 13–64% experience depression. [4]. Furthermore, some studies have highlighted frequent psychosomatic disorders, insecure attachment styles, traumatic experiences, and dissociative symptoms in FM patients [5,6,7,8]. 

Environmental factors such as stressful events, emotional and physical traumas, lack of social support, and certain individual characteristics may be associated with the onset of FM and a worsening of perceived pain and psychological distress [4]. Other individual aspects such as personality traits may influence both adaptation to chronic pain conditions and psychological distress, which are often associated with FM [4,9]. 

Indeed, several theoretical models have suggested that some personality characteristics lead to a worse response to stressors and adjustment to diseases in people with chronic pain conditions, such as patients with FM [4,9]. Some studies consider the personality model of Cloninger, which identifies the dimensions of temperament and characteristics as constitutive elements of personality [10]. This model includes four temperament traits that represent genetically determined aspects that are traceable in automatic emotional responses and three-character dimensions that are influenced by environment, experiences, and learning. Higher harm avoidance and lower self-directedness were observed in previous studies on patients with chronic pain [9]. Individuals with higher levels of harm avoidance are more fearful, pessimistic, and sensitive to criticism; lower levels of self-directedness suggest low self-esteem and low goal motivation [4,9]. Moreover, high self-transcendence and low cooperativeness levels have also featured in some FM patient studies [9]. Alexithymia is another personality dimension with a high prevalence in FM patients (between 48% and 68%) and leads to worse physical and psychological health outcomes [7,11]. The evidence shows a strong relationship between alexithymia and depressive symptoms [12,13]. Horta-Baas and colleagues [12] hypothesized that alexithymia may worsen depressive symptoms due to the misinterpretation of body sensations. Alexithymia is defined as a “personality construct that reflects a deficit in the cognitive processing and regulation of emotional states” [14]. Specifically, it is conceptualized as having difficulty identifying and differentiating emotions and integrating them within already present cognitive structures [14]. Although some previous theories have hypothesized a definition of alexithymia as a defense mechanism [15,16], further studies have found associations between alexithymia and defense mechanisms, concluding that alexithymia is a separate construct that is conceptually related to defensive mechanisms [17,18].

Defense mechanisms are automatic processes that reduce conflicts and mitigate the effects of changing external and internal reality, modify the conscious experience, and regulate an individual’s emotional response. Defense mechanisms are classified by their maturity level and adaptive value, and their strict use corresponds with physical and psychological consequences [19].

Regarding physical consequences, defense mechanisms are undoubtedly one of the most important personality factors influencing people’s perceptions of pain.

It is well known that Freud [20] considered pain as the result of conversion neuroticism and believed that it had some kind of conflict resolution function. More generally, the classical psychoanalytic perspective assumes that somatic symptoms result from a shift of psychic energy from the emotional charge of mental processes to that of somatic innervations to express the derivatives of repressed forbidden impulses in a distorted way [21]. In other words, when a person has some immature defenses, they cannot be protected from disturbing and painful factors, and these factors show themselves in the form of various illnesses, such as psychosomatic disorders. 

Regarding psychological consequences, a previous meta-analysis highlighted the relationship between psychological disorders and the increased use of maladaptive defense mechanisms. In particular, depression was associated with high scores in immature defense styles [22].

Living with FM is a severe psychological distress, and it is likely that the patient’s predominant defense style may determine her or his psychological response. The response results from the activation of a defense mechanism, and it is also associated with the patient’s capacities to cope with health stressors.

In spite of the high prevalence of psychological distress reported by FM patients [3,4], only a small number of studies have investigated the role of defense mechanisms in the development and maintenance of psychological distress in FM patients [23,24]. 

In light of the previous literature, the main aim of our study was to investigate personality traits and defense mechanisms in a sample of FM patients and to compare them to a healthy control group (HC). In addition, we investigated the effect of personality traits and defense mechanisms on psychological distress (anxiety and depression symptoms) in both FM and HC groups. 

## 2. Materials and Methods

### 2.1. Participants and Procedure

Fifty-four patients with FM who were consecutively enrolled in the Città della Salute e della Scienza University Hospital of Turin were included in our study. Due to the high prevalence of FM in women and the decision to exclude sex-related effects, we only included women. An expert rheumatologist diagnosed FM in all of the included patients according to the diagnostic criteria of the American College of Rheumatology [1]. During a separate session, the subjects completed psychological scales after clinical and psychological interviews that assessed their sociodemographic and clinical characteristics.

We recruited fifty-four healthy women, balanced for age and educational level, from different social and cultural backgrounds in a community sample in Turin. Participants filled in paper-and-pencil questionnaires during a face-to-face meeting. 

Participant recruitment took place before the COVID-19 pandemic. 

The exclusion criteria for both groups were as follows: being younger than 18, having a low educational level (<5 years), lacking fluency in Italian, and a history of neurological or severe psychiatric disorders. 

The hospital ethics committee approved the study in accordance with the Declaration of Helsinki. All participants provided written informed consent to participate.

### 2.2. Measures

Sociodemographic, clinical information, and psychological variables

We asked the participants to provide sociodemographic information (i.e., age, educational level, marital status, and occupation), clinical information (i.e., duration and severity of illness), and psychological variables (personality traits, alexithymia, anxiety, and depressive symptoms).

In addition, patients with FM completed the “Pain” item from the Italian version of the Revised Fibromyalgia Impact Questionnaire (FIQ-R) [25,26] to assess the average pain intensity in the previous week on a scale of 0 to 10.

Defense Mechanisms

The Defense Style Questionnaire (DSQ) [27,28] is an 88-item instrument assessing four types of defenses, called defensive styles (Maladaptive; Image-Distorting; Self-Sacrificing; Adaptive). Conceptualized along a continuum of adaptiveness/maladaptiveness, the response categories range from 1 (strongly disagree) to 9 (strongly agree). The maladaptive style includes several defenses, such as projection, acting out, regression, passive-aggressive behavior, withdrawal, somatization, and inhibition; individuals who make greater use of this style show a basic inability to deal with their impulses and stressors by taking constructive action on their own behalf. The defenses included in this style are action-oriented. The image-distorting style includes defenses such as omnipotence-devaluation, splitting, and primitive idealization; compared to the previous style, these are image-oriented defenses, and individuals who have recourse to them tend to divide their image of self and the image of others according to extreme categories (e.g., good/bad, strong/weak), avoiding a reality that might be too stressful. The self-sacrificing style includes defenses such as reaction formation, pseudo-altruism, and inhibition; the defenses included in this style reflect a need to perceive oneself as kind, helpful to others, never angry, and those who use them tend to deny their own needs. Finally, the adaptive style covers defenses such as task orientation, affiliation, humor, suppression, and sublimation; these defenses allow distraction and reaction to distressing feelings and stressful situations by engaging in constructive actions and behavior, humor, or channeling feelings into creative activities. Higher scores indicate the higher use of the defense mechanism. Internal consistency reliability ranged from 0.57 to 0.85 for the main DSQ scales [28].

Temperament and Character Traits

The Temperament and Character Inventory—Revised (TCI–R) [10] is a self-report instrument that assesses personality according to the Cloninger multidimensional and psychobiological model. It is made up of 240 items on a five-point Likert scale ranging from 1 (definitively false) to 5 (definitively true). The TCI-R contains seven domain scales: four temperament scales (i.e., novelty seeking (NS), harm avoidance (HA), reward dependence (RD), and persistence (P)) and three-character scales (i.e., self-directedness (SD), cooperativeness (C), and self-transcendence (ST)). Particularly, NS indicates a tendency towards active curiosity for novelty, impulsive decision-making, and active avoidance of monotony; HA reflects a tendency to be shy and worried in anticipation of possible danger; RD reveals a tendency for sentimentality, social attachment, and dependence on the approval of others; P indicates a tendency for ambition and perseverance despite frustration, whereas SD describes the ability to control and regulate personal behavior in accordance with chosen goals and values; C is reflected in an inclination for social tolerance, empathy, availability, and compassion; ST indicates a tendency to be more spiritual [10]. For this study, we used the Italian version of the TCI-R [29]. Internal consistency reliability ranged from 0.79 (RA) to 0.91 (persistence).

Alexithymia

The Toronto Alexithymia Scale (TAS-20) [30,31] is a self-report instrument. It includes 20 items, each with responses recorded on a five-point Likert-type scale, according to the agreement with the statements. Item scores yield three subscale scores and a composite score [32]. The subscales assess the different aspects that characterize alexithymia: “difficulty identifying feelings” (i.e., the inability to distinguish among emotions and between them and the bodily sensation of arousal), “difficulties describing feelings” (i.e., the inability to verbalize one’s emotions), and externally oriented thinking (i.e., the difficulty in focusing on internal emotional experience) [30,31]. This scale demonstrates good internal consistency (Cronbach’s α ≥ 0.70) and test–retest reliability [31].

Psychological distress

The Hospital Anxiety and Depression Scale (HADS) [33,34] is a self-report instrument that is used to assess depression and anxiety. It includes 14 items that represent two subscales: anxiety (HADS-A) and depression (HADS-D). This questionnaire excludes references to somatic symptoms, thus avoiding an overlap of symptoms between somatic disease or syndrome and mood disorders [35]. The total score can range from 0 to 21, and a score of 8 or higher is indicative of clinically significant levels of depression or anxiety. Taken together, a high score (cut-off > 15) provides a general indication of psychological distress. The HADS demonstrates good concurrent validity, test–retest reliability, and internal consistency (Cronbach’s α = 0.82–0.90) [27]. 

### 2.3. Statistical Analyses

Independent *t*-tests and the Mann–Whitney U-test for independent samples were used to evaluate possible differences in the sociodemographic and psychological variables (personality traits, defense mechanism, and psychological distress), respectively, between the FM and HC groups. Finally, we ran two hierarchical multiple regression analyses to assess whether clinical variables were significant predictors of psychological distress (HADS total score) in both the FM and HC groups. In both the regression models, the independent variables were entered as follows: temperament and character traits (TCI) were entered in the first block, defense mechanisms (DSQ) were entered in the second block, and alexithymia (TAS) was entered in the third block. To avoid unnecessary reductions in statistical power, independent variables were only included when they significantly correlated with the dependent variables. We assessed collinearity using the statistical factors of tolerance and the variance inflation factor. All analyses were performed using the Statistical Package for Social Sciences version 26 (SPSS-26; IBM. Armonk, NY, USA). 

## 3. Results

The FM patients had a mean age of 49.9 (10.9) years and an average education level of 13 (3.3) years. The HC participants had a mean age of 51.6 (5.7) years and an average education level of 14.5 (3.1) years. Most participants in both the FM and HC groups were married (53.7% and 75.9%, respectively) and employed (63% and 74%, respectively) (Table 1).

The results of the *t*-tests revealed that patients with FM and the HC group were balanced in terms of age and educational level. Regarding the clinical characteristics of the FM group, patients who had had their illness for an average of 9 years reported high pain intensity (FIQ-R Pain: 6.9 ± 2.1). Regarding temperament and character traits, the Mann–Whitney U-test for independent samples showed that the FM patients obtained significantly higher scores on the TCI-HA (U = 347; z = −6.828; *p* < 0.001) and lower scores on the TCI-SD (U = 1096; z = 2.753; *p* < 0.003) and TCI-P (U = 2096; z = 3.922; *p* < 0.001) compared to the HC group. Further, regarding defense mechanisms, the FM patients obtained higher scores compared to the HC on the DSQ-M (U = 759.5; z = −4.295; *p* < 0.001). Finally, the FM patients obtained significantly higher scores on both the TAS (U = 873; z = −3.596; *p* < 0.001) and HADS (U = 719.5; z = −4.543; *p* < 0.001) (Table 2).

We ran two hierarchical multiple regression analyses to explore the relative contributions of personality traits and defense mechanisms on the HADS total score in both the FM and HC groups. In the FM group, the full regression model significantly predicted the HADS total score, F (1, 47) = 9.222, *p* = 0.004, adj. R^2^ = 0.48. Among all of the predictors, alexithymia (TAS-tot) (β = 0.426, *p* = 0.004), harm-avoidance (TCI-HA) (β = 0.299, *p* = 0.014), and maladaptive defense style (DSQ-M) (β = 0.393, *p* = 0.015) were statistically significant (Table 3).

In the HC group, the full regression model significantly predicted the HADS total score, F (2, 50) = 5.008, *p* = 0.004, adj. R^2^ = 0.18. Among all of the predictors, harm-avoidance (TCI-HA) (β = 0.344, *p* = 0.029) and the adaptive defense style (DSQ-A) (β = 0.105, *p* = 0.019) were statistically significant (Table 4).

## 4. Discussion

The first aim of our study was to investigate both the personality traits and defense mechanism styles in FM patients in comparison with the HC.

The main results show that FM patients showed higher alexithymia, higher harm avoidance, lower self-directedness, and lower persistence compared with the HC. These findings, in line with previous studies [4,9,36,37,38], suggest a tendency for FM patients to worry and fear, be sensitive to criticism and punishment, and be pessimistic. Moreover, they are more likely to be destructive, fragile, and lacking an internal locus of control, with low self-efficacy and self-esteem. Some see themselves as unstable, inactive, and would often do anything to delay starting a job. Finally, they are powerless in identifying, recognizing, and expressing their own feelings and those of others [11]. FM patients tend to use maladaptive defense style more than HC. This indicates that they face stressful events using immature mechanisms such as projection, somatization, acting out, and regression more often than HC. This last result is in contrast to a previous study [23] that found no difference in defense mechanisms between FM and HC groups.

Our second aim was to evaluate the effect of personality traits and defense mechanisms on psychological distress in both FM and HC groups. The main results suggest that alexithymia, harm avoidance, and maladaptive defense style are significant predictors of psychological distress in FM patients and that the final model explained 48% of the variance. Our results are in line with previous studies that have already shown the role of harm avoidance [39], alexithymia [13], and immature mechanisms [23] in psychological distress in FM patients. Moreover, the relationship between immature defense mechanism style and psychological distress is present in other clinical populations [40,41,42], and several studies have shown that maladaptive defense styles were associated with major depression [43] and with the presence and severity of patient-reported depression symptoms [44].

Within the maladaptive style is also the mechanism of somatization, on which it seems useful to dwell. The association between somatization defense and distress is also in line with the theoretical model on which the DSQ has been constructed. Indeed, the DSQ assesses somatization defense by directly linking the appearance of bodily symptoms with psychological distress (e.g., “I get physically ill when things aren’t going well for me”), thereby assuming that a defensive conversion of psychic derivatives into bodily symptoms is activated [45].

However, the study of Pahlevan and colleagues [46] proposed an interesting model of pain perception, through which they link avoidance behavior, defense mechanisms, and alexithymia. Avoidance behavior is a typical behavior pattern in people with chronic pain, and it is caused by the immaturity of their defenses and alexithymia, and it causes the higher intensity of pain perception.

It is well known that pain intensity is one of the most stressful symptoms that patients with FM sufferers face and that involves high levels of psychological distress [47].

Since the focus of the present study was the identification of predictors for psychological distress in FM patients, it was important to distinguish the factors that might be associated with psychological distress in healthy people from those that might be associated with distress among the FM patients.

As for the HC group, the results showed that harm avoidance and adaptive defense style are significant predictors of psychological distress.

Regarding temperamental traits, our findings are in line with previous studies [48,49,50,51] that showed the positive association between harm avoidance and psychological distress among healthy subjects. Moreover, a previous meta-analysis [52] took longitudinal general population sample studies that supported the hypothesis that HA is a susceptibility factor for depression into consideration. Indeed, in accordance with the Cloninger’s model [53], HA is the temperament dimension that corresponds to behavioral inhibition, and it is related to serotonergic function.

In summary, despite the fact that HA levels are higher in FM than they are in HC, this temperamental trait seems to be associated with psychological distress both in general and in clinical populations.

Regarding defense mechanisms, our findings are surprising because they are in contrast with those from previous literature that highlight the protective and positive role of mature defense styles in the greater psychological wellbeing in the general population [54]. One possible interpretation of our results could be that these adaptive defense mechanisms (e.g., task orientation, affiliation, humor, suppression, and sublimation), although conceptualized as mature strategies, can become unhelpful if they are used excessively for a long period of time or in a rigid way. Using these defenses on a long-term basis could lead people to override difficult feelings, which may contribute to their poor well-being.

Taken together, our results suggest that FM patients show specific personality traits and are characterized by the significant use of specific immature defense mechanisms. Moreover, both personality traits (alexithymia and harm avoidance) and maladaptive defense mechanism style partly explain the presence of anxiety and depressive symptoms in FM patients.

The literature about a specific personality profile in FM is controversial [55], likely due to using different theoretical models of personality and different measures across studies. However, both personality factors [56] and defense mechanisms [57] may partly explain the variation by enhancing vulnerability to anxiety and depressive symptoms.

The present study also has some limitations. First, the study used self-report questionnaires. Second, we adopted a cross-sectional design, which does not allow us to draw concrete conclusions about the causality of the emergent relationships. Finally, as our study was conducted on a small sample, our findings should be considered with caution.

Despite these limitations, the present study is the first to explore the contribution of both defense mechanisms and personality characteristics on psychological distress among female FM patients in comparison to HC women. In relation to the biopsychosocial model [58], our findings have important clinical implications both in order to place greater emphasis on the psychological components of FM for medical practitioners and to define an increasingly targeted psychological diagnosis and treatment for psychotherapists.

In particular, with reference to the psychological diagnosis, the evaluation of personality traits needs to be taken into consideration when providing an effective diagnosis. Moreover, Vaillant [45] has argued that no mental status or clinical examination should be considered complete without identifying the patient’s predominant defense style. Indeed, understanding the patient’s individual personality and defense style allows the possibility of interpreting and treat the person beyond the symptom and can influence the therapist’s focus and style of intervention [59].

Specifically, this means that psychotherapists should pay more attention to temperamental and character traits, levels of alexithymia, and each patient’s own defensive style. It is well known that both alexithymia [60] and immature defense mechanisms [57] are negative predictors of psychological treatment outcome. A psychological treatment based on improving the processes of emotional regulation, making defense mechanisms more adaptive and flexible, and acting on character traits, allows patients with FM facing emotionally stressful situations to better manage pain and relationships and therefore decrease levels of psychological distress.

Although there is strong literature suggesting the effectiveness of cognitive behavioral treatment on pain and psychological distress in FM [61], by pointing out the impact that personality traits and defense mechanisms have on anxiety and depressive symptoms [62], long-term psychodynamic psychotherapy can be also proposed to improve the mental health of FM patients.

## Figures and Tables

**Table 1:** Sociodemographic and clinical characteristics of the FM patients and HC. Mean (SD), percentage, and *t*-test are listed.

	FM (*n* = 54)	HC (*n* = 54)	*t* Test (df)	*p*
Age (years)	49.9 (10.9)	51.6 (5.7)	−1.028 (80.42)	0.307
Educational level (years)	13.4 (3.3)	14.5 (3.1)	−1.870 (106)	0.064
Marital status				
Single	11 (20.4%)	5 (9.3%)		
Cohabiting	7 (13%)	2 (3.7%)		
Married	29 (53.7%)	41 (75.9%)		
Divorced	5 (9.3%)	5 (9.3%)		
Widowed	2 (3.7%)	1 (1.9%)		
Work status				
Student	3 (5.6%)	0 (0%)		
Employed	34 (63%)	40 (74.1%)		
Unemployed	6 (11.1%)	2 (3.7%)		
Housewife	6 (11.1%)	10 (18.5%)		
Retired	5 (9.3%)	2 (3.7%)		
Duration of illness (months)	112.80 (127.7)	–		
FIQ-R Pain	6.9 (2.1)	–		

FM = fibromyalgia; HC = healthy controls; df = Degrees of freedom; FIQ-R = Fibromyalgia Impact Questionnaire Revised version.

**Table 2:** Mean ranks for personality traits and defense mechanisms in FM vs. HC.

	FM (*n* = 54)	HC (*n*= 54)	*p*
Temperament and Character Traits			
TCI-NS	54.12	54.48	0.900
TCI-HA	75.07	33.93	<0.001
TCI-RD	53.86	55.14	0.832
TCI-P	42.69	66.31	<0.001
TCI-SD	46.20	62.80	0.006
TCI-C	52.32	56.68	0.470
TCI-ST	53.01	55.99	0.621
Alexithymia			
TAS-TOT	65.33	43.67	<0.001
Defense Mechanisms			
DSQ-A	50.78	58.22	0.217
DSQ-M	67.44	41.56	<0.001
DSQ-ID	52.33	56.67	0.472
DSQ-SS	54.52	54.48	0.995
Psychological distress			
HADS-TOT	68.18	40.82	<0.001

FM = fibromyalgia; HC = healthy controls; df = degrees of freedom; TCI = Temperament and Character Inventory—Revised: TCI-NS = novelty seeking; TCI-HA = harm avoidance; TCI-RD = reward dependence; TCI-P = persistence; TCI-SD = self-directedness; TCI-CO = cooperativeness; TCI-ST = self-transcendence; DSQ = Defense Style Questionnaire: DSQ-A = DSQ-adaptive style; DSQ-M = DSQ-maladaptive style; DSQ-ID = DSQ-image-distorting; DDSQ-SS = SQ-self-sacrificing style; TAS-TOT = Toronto Alexithymia Scale-20 total score; HADS-TOT = Hospital Anxiety and Depression Scale total score.

**Table 3:** Hierarchical multiple regression predicting HADS total scores from temperament and character traits, defense mechanisms, and alexithymia in the FM group (N = 54).

HADS-TOT								
Predictors	B	β	t	95% CI	Adj R^2^	F	ΔR^2^	ΔF
**Model 1**					0.331	9.738 ***	0.369	9.738 ***
TCI-HA	0.275	0.505	4.268 ***	0.146; 0.405				
TCI-SD	−0.070	−0.234	−1.848	−0.145; −0.006				
TCI-CO	−0.032	−0.055	−0.418	−0.187; 0.122				
**Model 2**					0.393	7.877 ***	0.082	3.579 *
TCI-HA	0.266	0.415	3.482 **	0.096; 0.357				
TCI-SD	−0.032	−0.107	−0.814	−0.110; 0.047				
TCI-CO	0.015	0.026	0.191	−0.142; 0.172				
DSQ-M	0.215	0.384	2.285 *	0.026; 0.404				
DSQ-ID	−0.057	−0.036	−0.220	−0.574; 0.461				
**Model 3**					0.482	9.222 ***	0.090	9.210 ***
TCI-HA	0.163	0.299	2.563 *	0.035; 0.291				
TCI-SD	−0.008	−0.026	−0.211	−0.082; 0.067				
TCI-CO	0.012	0.020	0.160	−0.134; 0.157				
DSQ-M	0.220	0.393	2.532 *	0.045; 0.395				
DSQ-ID	−0.415	−0.261	−1.563	−0.949; 0.119				
TAS-TOT	0.228	0.426	3.035 **	0.077; 0.380				

TCI = Temperament and Character Inventory—Revised: TCI-HA = harm avoidance; TCI-SD = self-directedness; TCI-CO = cooperativeness; TCI-NS = novelty seeking; DSQ = Defense Style Questionnaire: DSQ-M = maladaptive style; DSQ-ID = image-distorting; TAS-TOT = Toronto Alexithymia Scale-20 total score; CI = confidence interval; * *p* < 0.05; ** *p* < 0.01; *** *p* < 0.001.

**Table 4:** Hierarchical multiple regression predicting the HADS total scores from temperament and character traits, defense mechanisms, and alexithymia in the HC group (N = 54).

HADS-TOT								
Predictors	B	β	t	95% CI	Adj R^2^	F	ΔR^2^	ΔF
**Model 1**					0.103	7.087 **	0.120	7.087 **
TCI-HA	0.149	0.346	2.662 **	0.037–0.261				
**Model 2**					0.185	5.008 ***	0.111	3.613 *
TCI-HA	0.148	0.344	2.252 *	0.016–0.280				
DSQ-A	0.253	0.105	2.417 *	0.043–0.463				
DSQ-M	0.039	0.093	0.421	−0.147–0.225				

TCI = Temperament and Character Inventory—Revised: TCI-HA = harm avoidance; DSQ = Defense Style Questionnaire: DSQ-M = maladaptive style; DSQ-A = adaptive style; CI = confidence interval; * *p* < 0.05; ** *p* < 0.01; *** *p* < 0.001.

## Data Availability

The data presented in this study are available upon request from the corresponding author. The data are not publicly available due to compliance with privacy laws.
